# Trends in maternal oral health services at primary healthcare centers in Saudi Arabia: a cross-sectional study

**DOI:** 10.1186/s12903-023-03712-6

**Published:** 2023-12-21

**Authors:** Dania Al Agili

**Affiliations:** Department of Dental Public Health, King Abdulaziz University, P.O. Box 80200, 21589 Jeddah, Saudi Arabia

**Keywords:** Oral health, Pregnancy, Primary health care, Oral health services, Trends, Saudi Arabia

## Abstract

**Background:**

During pregnancy, many complex physiological changes and increased levels of pregnancy hormones are associated with adverse oral health and increased prevalence of periodontal disease. Our study aimed to assess the oral health needs of pregnant women and describe the patterns of dental services provided to them before, during, and after pregnancy. Assessing the oral health needs of pregnant women and understanding the patterns of dental services provided to them are important to facilitate efficient utilization of oral health services to promote better health outcomes for the mother and baby.

**Methods:**

Our study utilized a cross-sectional design to examine the prevalence of dental problems and use of dental services among a sample of postpartum women who visited primary healthcare centers (PHCs) in Jeddah for antenatal care, between 2018 and 2019. A link to a questionnaire adapted from the Pregnancy Risk Assessment Monitoring System (PRAMS) was sent to participants via the WhatsApp messaging platform. A total of 1350 postpartum women responded to the online survey. We estimated the prevalence of dental problems among women before and during pregnancy and assessed the association between their dental problems and their respective demographic characteristics. We calculated the prevalence of each dental service received before, during, and after pregnancy and examined the trends in dental services over these three periods. All bivariate associations were tested using Pearson’s chi-squared test.

**Results:**

We found that significantly fewer women visited a dental clinic during pregnancy (31.0%) compared to pre-pregnancy (38.2%) and post-pregnancy (47.3%). The prevalence of toothache, dental caries, gum disease, and dental extraction need before pregnancy was 45.9%, 57.0%, 27.3%, and 40.0%, respectively. These percentages remained the same during pregnancy, except for the need for dental extraction, which significantly decreased to 35.3%. Check-up dental visits increased significantly to 70.6% during pregnancy compared to pre-pregnancy (51.7%) and post-pregnancy (59.9%).

**Conclusion:**

Increasing women’s awareness of the importance and safety of oral healthcare during pregnancy, training dental students and primary healthcare dentists in the practice guidelines for the dental management of pregnant women, and developing and monitoring key performance indicators for maternal oral healthcare are the starting steps for improving the oral health and well-being of women and their children.

## Background

Pregnancy is a unique experience for women. During pregnancy, many complex physiological changes occur that can adversely affect oral health [1]. Many studies have reported that the oral healthcare needs of pregnant women are completely different from those of non-pregnant women [2–5]. The increased levels of pregnancy hormones, namely progesterone and estrogen, affect dental health and are associated with an increased prevalence of periodontal disease during pregnancy. Fluctuations in these hormones increase capillary permeability in the gingiva of pregnant women and enhance the production of prostaglandins resulting in an exaggerated inflammatory response of the periodontium to dental plaque [1, 6]. Gingivitis is the most common form of oral disease affecting pregnant women internationally [7–10]. Nearly half of the women with preexisting gingivitis endure significant exacerbation during pregnancy, which, if left untreated, could lead to tooth loss [11, 12]. In addition, the increased acidity in the mouth associated with early morning sickness, acidic and sugary dietary cravings, and inadequate oral hygiene during pregnancy all increase women’s risk of dental erosion and dental caries [11, 12].

The oral health of pregnant women can affect their overall health and the health of their unborn child [1]. Pregnant women with untreated periodontal disease and oral infections are at an increased risk of developing sepsis, preeclampsia, miscarriages, and adverse birth outcomes such as preterm birth and low birth weight [13–20]. Several systematic reviews reported odds ratios in the range of 2 to 4 between periodontal disease and the risk of preterm birth, low birth weight, and/or preterm low birth weight outcome. However, most of these reviews concluded that further randomized clinical trial studies with sufficient follow-up period are needed to validate these associations [14, 15, 18, 20, 21]. Furthermore, the transfer of mutans streptococci (MS**)** from mothers to infants has been recognized as a pathway by which children are initially inoculated with MS. The early acquisition of MS increases the risk of developing early childhood caries (ECC) in children. ECC is a serious oral disease associated with local pain, infection, and abscesses that compromise the children’s well-being and quality of life [22]. Despite the importance of maintaining good oral health for the health and well-being of both the pregnant woman and her unborn child, half of pregnant women globally endure dental pain during pregnancy [22].

Several reports on pregnant women in Saudi Arabia indicate that more than one-third of pregnant women endure dental infections and pain during pregnancy, and up to 60% of pregnant women suffer from periodontal disease [23–25]. The 2017 Saudi Arabian National Demographic and Health Survey (KSADHS) reported that 69% of women presented pain or problems in their teeth, or gums, or mouth [26]. Most pregnant women (70%–90%) in different regions in Saudi Arabia reported having at least one dental problem that requires dental care and attention; however, less than half of them receive the care they need [23, 24, 27, 28]. Poor awareness of the importance of good dental care during pregnancy and the negative perception about the safety of dental treatment during pregnancy are among the common reasons why pregnant women avoid dental care [27, 28].

Most studies on the utilization of dental care services during pregnancy only focus on the overall percentage of women who visit a dentist during pregnancy. Other reports measure the prevalence of women who received a dental cleaning during pregnancy. For example, an analysis of the PRAMS datasets of 75,876 American women between 2012 and 2015 showed that 51.7% of American pregnant women had at least one dental cleaning visit during their most recent pregnancy [29]. Although these measures are important to examine access to dental care for pregnant women, they do not estimate the prevalence of other dental services provided to them and/or indicate if these pregnant women received the dental treatment they truly needed. Many pregnant women visit the dental clinic during pregnancy but do not receive the needed dental care. Our study not only reports on the prevalence of an array of dental services rendered to women before, during and after their pregnancy, but it also shows how these services vary in prevalence and pattern over these three periods.

## Methods

### Study design and scope

This cross-sectional descriptive study examined the utilization of dental services by women who visited primary healthcare centers (PHCs) in Jeddah for antenatal care between 2018 and 2019. There are 46 PHCs associated with five major public general hospitals in Jeddah, Saudi Arabia. These PHCs provide free preventive and treatment services, including the management of chronic diseases, antenatal care, and dental services to Saudi nationals and other eligible individuals, such as spouses or children of Saudi nationals. Treatment includes the management of chronic diseases, antenatal care, and dental services.

### Study population and sample selection

Our study targeted women who attended antenatal care clinics between 2018 and 2019 at PHCs in Jeddah. The contact information of these women was obtained from the directors of the PHCs. The inclusion criteria for women in the study were as follows: (1) Women who had a pregnancy that ended in a live birth, (2) working mobile phone number, (3) Arabic language skills, and (4) consent to participate in the study. An active phone number was important to enable us to send the online survey via the WhatsApp messaging application. A total of 3024 women met the inclusion criteria. A random sample of 2026 women, which comprised two-thirds of the original population, was selected for this study. Since this was a relatively homogenous group, this selection adequately represented the target population without incurring the additional costs of including all 3024 women. The exclusion criteria were (1) incorrect phone numbers, (2) lack of Arabic language skills, (3) death of mother, and (4) pregnancy resulting in stillbirth or miscarriage. Upon applying these exclusion criteria, 1,792 women represented the final study sample. Upon attempting to contact these women, 307 did not answer calls or messages, and 79 refused to participate. We received 1406 responses, of which 56 were incomplete. Therefore, the final study sample included 1,350 women. A detailed description of the sampling technique and sample selection is provided in a previous paper [30].

### Study questionnaire

A structured quantitative questionnaire, adapted from the Pregnancy Risk Assessment Monitoring System (PRAMS), was developed in SurveyMonkey. A detailed description of the questionnaire’s design and development and its validity and reliability is provided in a previous paper [30]. The questionnaire assessed the participants’ oral health problems and dental service utilization at three time points: 1) 6 months before pregnancy, 2) during pregnancy, and 3) after pregnancy. The oral health problems examined in this study included toothache; tooth decay; red, swollen, or bleeding gums; need for extraction; and dental trauma. The various dental health services utilized in the perinatal period include (1) checkups, (2) prophylaxis, (3) fillings, (4) periodontal treatments, (5) extractions, (6) radiographs, and (7) “other services”. The “other” category was a free text field that allowed participants to enter the services they utilized and was not among the six options listed. The respondents’ demographic characteristics that we examined included age (categorized as < 20 years, 20–29 years, 30–39 years and 40 + years), highest educational attainment (< 12 years of education, 12 years of education, and > 12 years of education), family monthly income in Saudi Arabian Riyals (SAR) (≤ 5000, 5001–7000, 7001–10000, and > 10,000 SAR [$1 = 3.75 SAR]), and medical insurance status (whether or not they had dental coverage), and nationality (Saudi Arabian or non-Saudi Arabian).

### Data collection

Data collection began in February 2021 and concluded in January 2022. An electronic link to the survey was sent to the participants via WhatsApp. A statement describing the study purpose, procedures, voluntary participation, confidentiality, and contact information of the principal investigator was also sent to the participants along with the survey link. The actual submission of the online survey indicated participants’ consent. We made several calls to participants who did not respond to the WhatsApp message on different days of the week and at different times of the day to ensure that we reached as many participants as possible.

### Ethical clearance

King Abdulaziz University Faculty of Dentistry Research Ethics Committee [070–07-20] and the Institutional Review Board at the Ministry of Health [20-591E] provided the ethical approval for this study. Additionally, research clearance was obtained from the directors of the research centers in each of the five public general hospitals. The study was performed in accordance with all the relevant guidelines and regulations.

### Statistical analysis

We calculated the prevalence of each oral health problem (toothache; tooth decay; red, swollen or bleeding gums; need for extraction; and dental trauma) among women before and during pregnancy and assessed its association with each demographic characteristic (age, education, family income, health insurance, and nationality). We also calculated the prevalence of each dental service received by women before, during, and after pregnancy and examined the trends in dental services over these time periods. These services included checkup, prophylaxis, fillings, periodontal treatment, dental extractions, radiographs, and other services. We categorized the free text provided by the respondents who chose the option “other service” and examined their prevalence during these three periods. Associations between dental problems and participants’ characteristics before and during pregnancy were tested using Pearson’s chi-squared test. Similarly, associations between dental problems before and during pregnancy and associations between type of dental service before, during, and after pregnancy were tested using Pearson’s chi-squared test. *P*-value was considered statistically significant at ≤ 0.05. SPSS Statistics for Windows Version 28.0.0.0 (IBM, Armonk, NY) was used for data analysis.

## Results

There were 1350 respondents in our study, representing a 75% response rate of the included sample (1,792). Of these respondents, 515 (38.15%) received dental care before conception, 419 (31.04%) during pregnancy, and 693 (47.33%) after pregnancy. Some respondents received oral healthcare during more than one period.

We found that 85.2% and 79.4% of the women had at least one dental problem before or during pregnancy, respectively. Table 1 shows the reported oral health problems before and during pregnancy by the demographic characteristics of women. Before and during pregnancy, the prevalence of tooth decay (59.7% and 56.7%, respectively) and the need for extraction (43.1% and 36.7%, respectively) were the highest in the 30–39 years-old group, whereas toothache (49.4% and 53.0%, respectively) was the highest in the 40 + group. In both periods, there was an inverse dose–response relationship between age and prevalence of dental trauma. In addition, as the level of women’s education increased, the prevalence of toothache, need for extraction, and dental trauma significantly decreased during the pre-pregnancy period. A similar statistically significant pattern was observed for the need for extraction during pregnancy. We also found that women in the lowest family income bracket had the highest prevalence of toothache (49.5%, *p* < 0.05)) before pregnancy. Those without health insurance had a significantly higher prevalence of tooth decay, gum problems, and need for extraction both before and during pregnancy, whereas for toothache, statistical significance was observed only before pregnancy.

**Table 1:** Prevalence of oral health problems before and during pregnancy by characteristics of women, *N* = 1350

Characteristic	Before Pregnancy					During Pregnancy				
	Toothache	Tooth decay	Red, swollen or bleeding gums	Need for extraction	Dental trauma	Toothache	Tooth decay	Red, swollen or bleeding gums	Need for extraction	Dental trauma
	N (%)	N (%)	N (%)	N (%)	N (%)	N (%)	N (%)	N (%)	N (%)	N (%)
Age group										
< 20	4 25.0%	6 37.5%	2 12.5%	5 31.3%	1 6.3%	7 43.80%	7 43.80%	3 18.80%	5 31.30%	2 12.50%
20–29	206 43.4%	266 56.0%	131 27.6%	170 35.8%	29 6.1%	230 48.40%	262 55.20%	132 27.80%	150 31.60%	35 7.40%
30–39	328 47.3%	414 59.7%	187 27.0%	299 43.1%	41 5.9%	343 49.50%	393 56.70%	185 26.70%	254 36.70%	45 6.50%
≥ 40	82 49.4%	84 50.6%	49 29.5%	71 42.8%	7 4.2%	88 53.00%	78 47.00%	40 24.10%	68 41.00%	9 5.40%
Education										
< 12 Years of education	133 56.8%^***^	142 60.7%	72 30.8%	116 49.6%^***^	22 9.4%^*^	132 56.40%	137 58.50%	66 28.20%	105 44.90%^***^	23 9.80%
12 Years of education	216 46.1%	261 55.7%	124 26.4%	195 41.6%	28 6.0%	229 48.80%	258 55.00%	115 24.50%	171 36.50%	30 6.40%
> 12 Years of education	271 41.9%	367 56.7%	173 26.7%	234 36.2%	28 4.3%	307 47.40%	345 53.30%	179 27.70%	201 31.10%	38 5.90%
Family income										
≤ 5000	284 49.5%^*^	320 55.7%	150 26.1%	242 42.2%	36 6.3%	299 52.1%	304 53.0%	156 27.2%	214 37.3%	45 7.8%
5001–7000	147 47.9%	188 61.2%	88 28.7%	117 38.1%	16 5.2%	150 48.9%	179 58.3%	77 25.1%	105 34.2%	18 5.9%
7001–10000	114 39.6%	164 56.9%	73 25.3%	110 38.2%	17 5.9%	134 46.5%	160 55.6%	73 25.3%	96 33.3%	19 6.6%
> 10000	75 41.4%	98 54.1%	58 32.0%	76 42.0%	9 5.0%	85 47.0%	97 53.6%	54 29.8%	62 34.3%	9 5.0%
Health insurance										
No	558 47.0%^*^	698 58.8%^***^	342 28.8%^***^	494 41.6%^**^	73 6.1%	591 49.7%	664 55.9%^*^	327 27.5%^*^	440 37.0%^***^	85 7.2%
Yes	62 38.3%	72 44.4%	27 16.7%	51 31.5%	5 3.1%	77 47.5%	76 46.9%	33 20.4%	37 22.8%	6 3.7%
Nationality										
Non-Saudi	73 48.0%	88 57.9%	38 25.0%	54 35.5%	8 5.3%	81 53.3%	76 50.0%	34 22.4%	52 34.2%	6 3.9%
Saudi	547 45.7%	682 56.9%	331 27.6%	491 41.0%	70 5.8%	587 49.0%	664 55.4%	326 27.2%	425 35.5%	85 7.1%
Total	620 45.9%	770 57.0%	369 27.3%	545 40.0%	78 5.8%	668 49.5%	740 54.8%	360 26.7%	477 35.3%	91 6.7%

Pearson’s χ^2^ test of significance: ^*^ *p* < .05; ^**^ *p* < .01; ^***^ *p* < .001

**Fig. 1:**
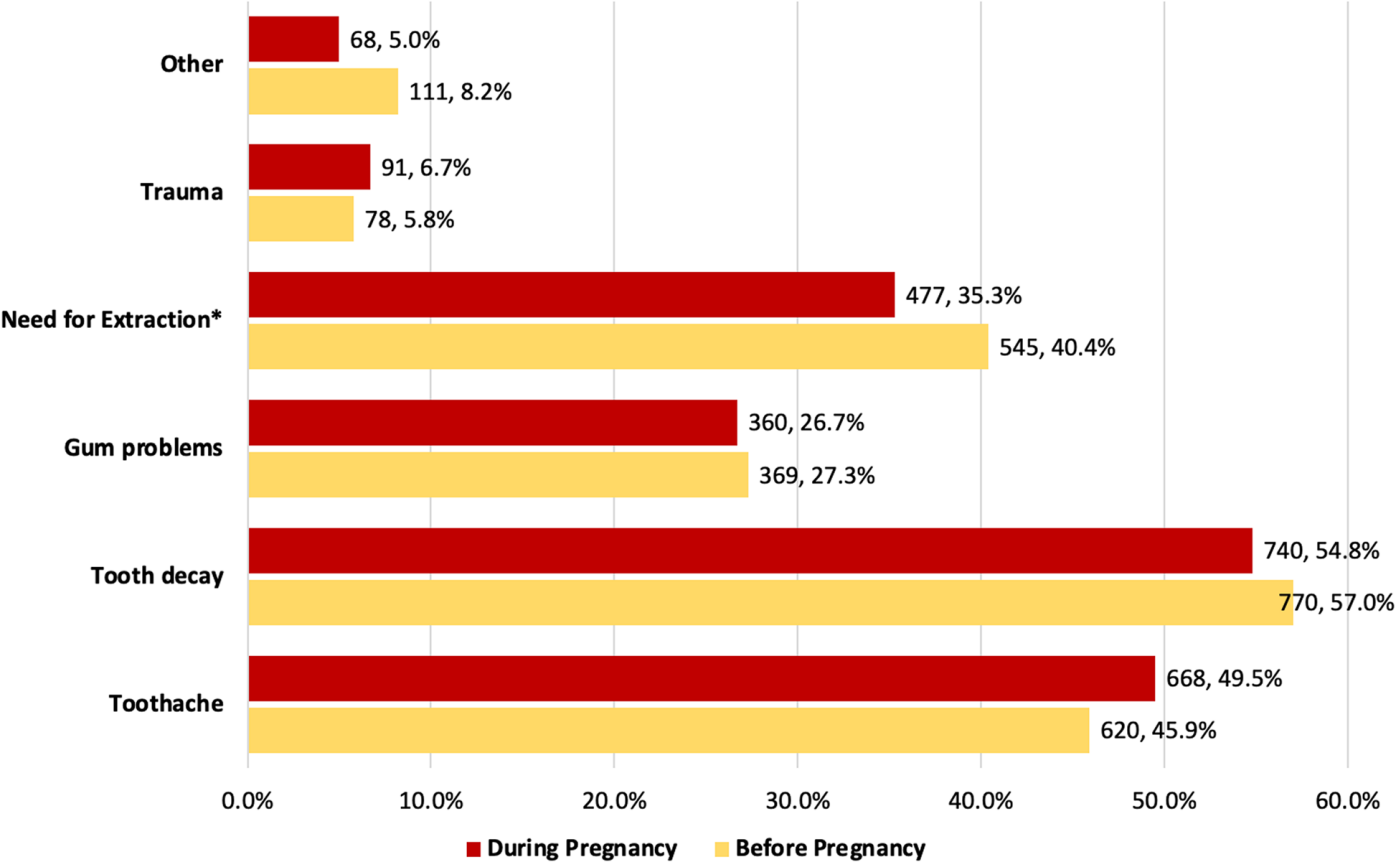
Prevalence of oral health problems among women before and during pregnancy, *N* = 1350. Pearson’s χ^2^ test of significance: * *p*-value < 0.01

Figure 1 shows the prevalence of oral health problems before and during pregnancy. The prevalence of all oral health problems remained the same before and during pregnancy, except for the need for dental extractions, which significantly decreased from 40.4% (*N* = 545) before pregnancy to 35.3% (*N* = 477) during pregnancy, *p* < 0.01.

**Fig. 2:**
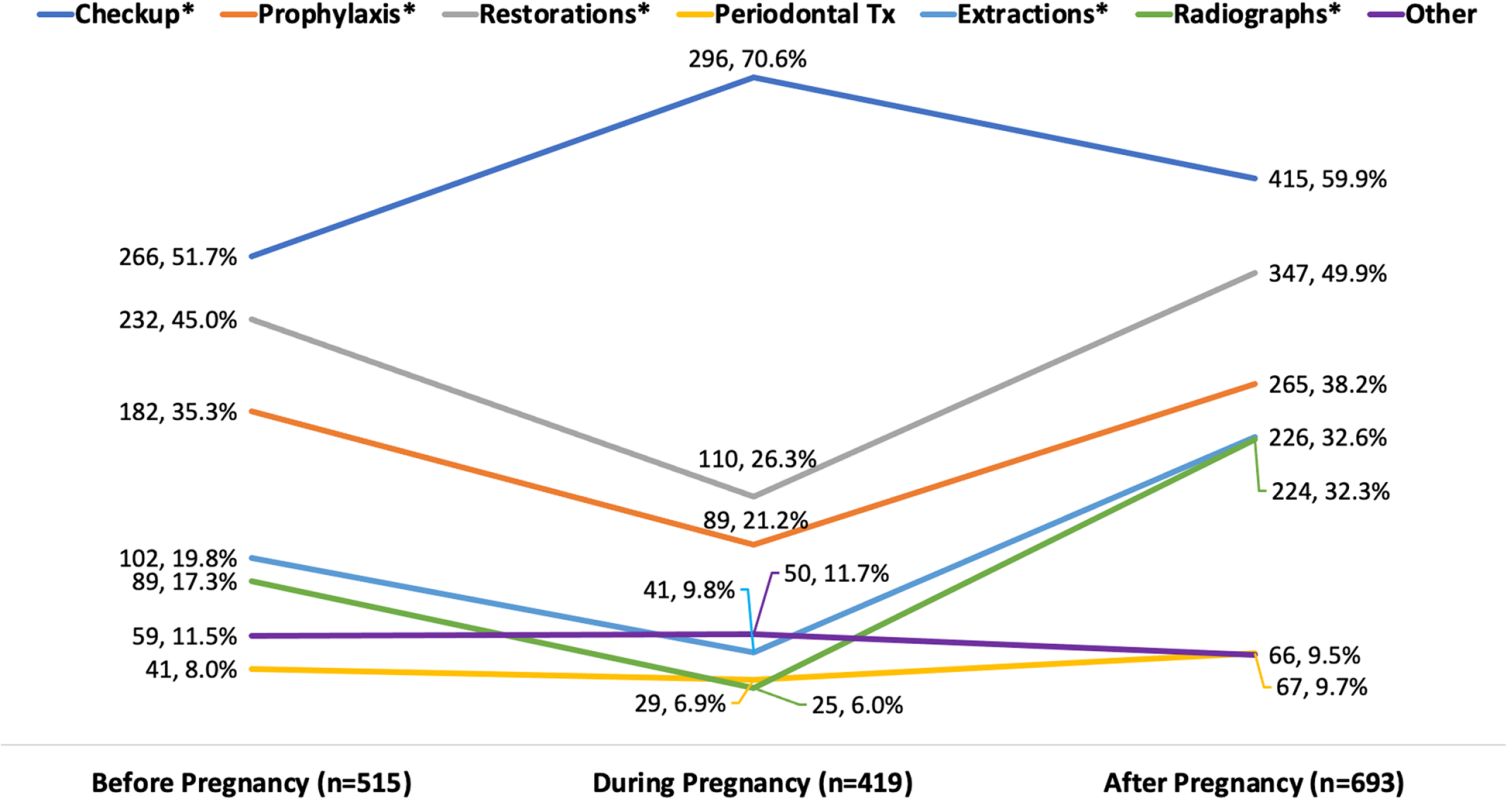
Dental services for women before, during, and after pregnancy. Pearson’s χ^2^ test of significance: * *p*-value < 0.001

Figure 2 shows the rates of oral health services utilized before, during, and after pregnancy. Among the seven possible options of service utilization given to the respondents, only checkups had a higher prevalence during the pregnancy period (70.6%) than during the pre-pregnancy (51.7%) and post-pregnancy periods (59.9%), *p* < 0.001. Radiography decreased the most from pre-pregnancy (17.3%) to pregnancy (6.0%) and then increased drastically in the post-pregnancy period (32.3%), *p* < 0.001. Similar patterns were observed for prophylaxis, dental restorations, and dental extractions. Periodontal treatment was the least used service by women before (8.0%), during (6.9%), and after pregnancy (9.7%). Free-form textual option which was categorized as ‘Other’ included endodontics, orthodontics, prosthodontics, implant dentistry, periodontics, oral surgery, cosmetic surgery, prescriptions, postponed treatments, and not specified. The ‘Other’ category had a prevalence of 8.0% before pregnancy, 6.9% during pregnancy, and 9.7% after pregnancy, but this change was not statistically significant.

**Fig. 3:**
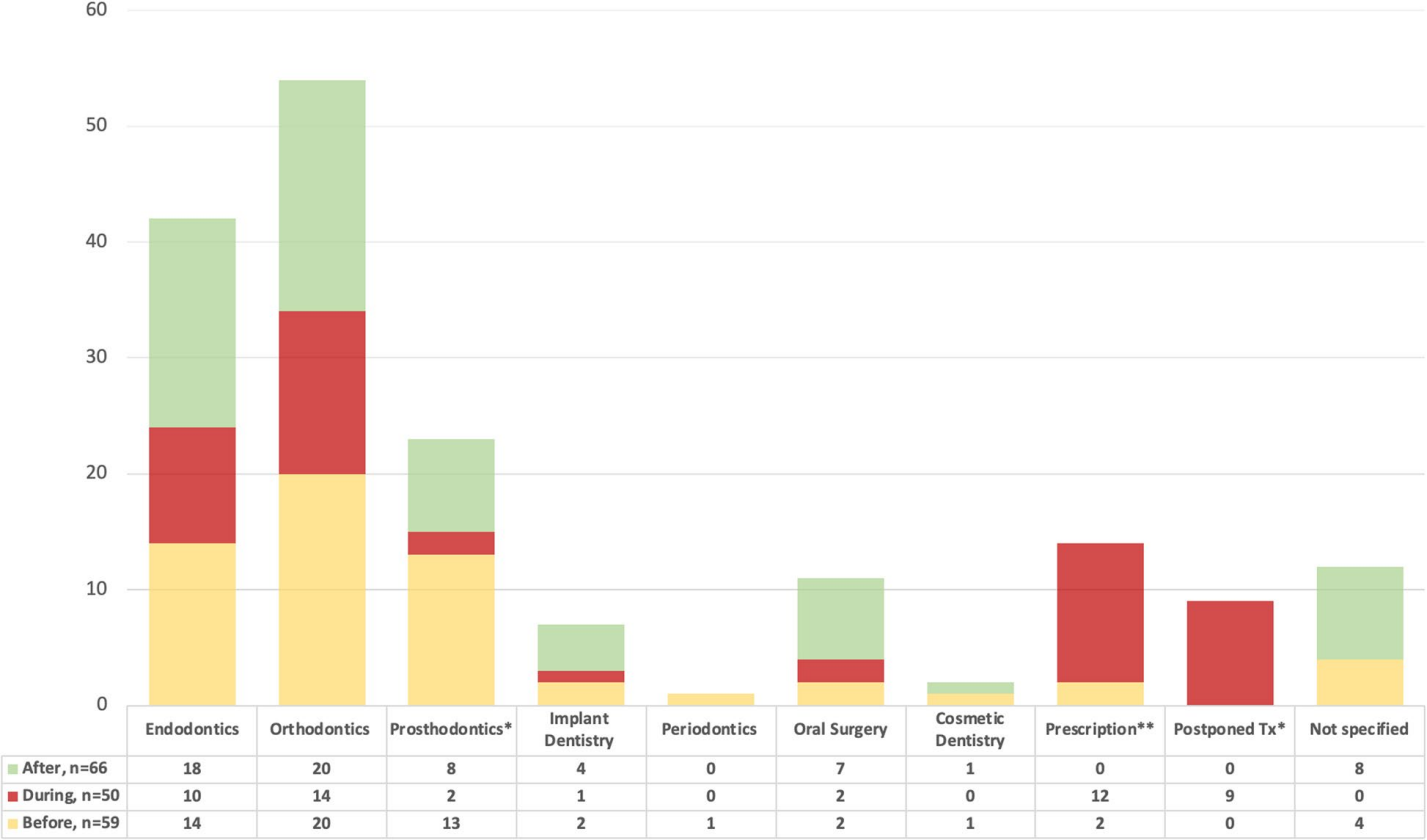
Frequency of “Other” dental services before, during, and after pregnancy. Pearson’s χ^2^ test of significance: * *P*-value < 0.05; ** *P*-value < 0.001

When we explored the “other services” utilized before, during, and after pregnancy (Fig. 3), prosthodontic service utilization decreased drastically (4%) during pregnancy and was higher before (22%) and after pregnancy (12%); whereas prescription (pain killers and antibiotics) increased in the pregnancy period (24%), as compared to the pre-pregnancy (3%) and post-pregnancy (0%) periods, and this association was statistically significant (*p* < 0.001). While dental treatment was postponed during the pregnancy period (18%), dental treatment was not postponed at all during the pre-pregnancy or the post-pregnancy periods. These associations were statistically significant, *p* < 0.05.

Finally, most of the participants received dental care before (80.4%, *N* = 414) and during (45.8%, *N* = 192) pregnancy through private dental practices. The proportions of women who received dental services at PHCs before and during pregnancy were 25.4% (*N* = 414) and 18.4% (*N* = 77), respectively. The remaining women received dental care before and during pregnancy at other government dental clinics (14.2%, *N* = 73 and 9.1%, *N* = 38, respectively).

## Discussion

Our study described trends in the utilization of dental services by women before, during, and after pregnancy. Our study found that despite the elevated number of dental problems exhibited by women during pregnancy, the prevalence of dental visits during pregnancy was significantly lower than that reported before and after pregnancy. We also found that despite the remarkable increase in dental check-up visits during pregnancy, all other dental services significantly declined.

Most studies have examined only the prevalence of dental visits during pregnancy. A cross-sectional study of Canadian women who visited a community health center for well-child visits reported that 93%, 80.5%, and 28.8% of these women received a dental check-up, preventive dental care, and dental or periodontal treatment, respectively, during pregnancy. Among those who reported receiving dental or periodontal treatment, fillings (32.5%) and extractions (10.0%) were the most frequent services [31]. Our study, however, reported the prevalence of dental services received during pregnancy relative to the need for such services. For example, about 27% of women reported having periodontal problems but only 21% and 6% received dental cleaning or periodontal treatment during pregnancy, respectively. Furthermore, at least 50% of women reported having toothache or tooth decay but only 26% received dental restorations during pregnancy. Therefore, there is a need to increase the rate of preventive and restorative dental services for our population of expectant mothers. Preventive dental visits should include dental evaluation, plaque removal, scaling and root planing, in addition to oral hygiene instructions.

Among the one-third of women who visited a dentist during pregnancy, most (71%) underwent dental checkups. This high percentage can be attributed to the successful implementation of a dental referral policy in PHCs. Prenatal health providers referred these women to the dental clinic for consultation, as per the PHC referral policy. However, the rate of all other dental services decreased significantly during pregnancy compared to that before pregnancy, while their dental problems remained the same as before pregnancy. Dental prophylaxis, a preventive dental service associated with a lower risk of preterm delivery [32], decreased significantly by 40% during pregnancy relative to the rate before pregnancy. Increasing the number of preventive dental visits during pregnancy is among the best practice criteria recommended by the Association of State and Territorial Dental Directors (ASTDD), which shows improved access to oral health care and/or improved oral health status of pregnant women [33]. Dental visits during pregnancy provide an opportunity for dentists to educate and perform dental treatment on expectant mothers who might otherwise be completely occupied after delivery with the care of their newborn and may find scheduling and attending dental appointments difficult [7].

Our findings also highlight the lack of attention paid to women’s periodontal health. Women with periodontal disease were not treated by the general dentists at the PHCs, nor were they referred to periodontists at the specialized dental centers. Further research is needed to explore the reasons for not referring these patients to the specialty centers to receive periodontal treatment. Successful treatment of periodontal disease early in the first trimester of pregnancy or even before becoming pregnant is safe for pregnant women and their unborn children; prevents adverse consequences of periodontitis for the mother, such as toothache and tooth loss; and may improve perinatal outcomes [34, 35].

Another alarming finding of this study was the pregnant women’s report of being prescribed pain medications and antibiotics when they presented to the dental clinic for toothache and/or dental infection, rather than being provided with the necessary dental care. Others were told to come after delivery to prevent potential harm to the fetus from X-rays, local anesthesia, filling material, and extractions. The American Academy of Periodontology has confirmed that the presence of an acute infection, abscess, or other potential sources of septicemia may necessitate prompt intervention, irrespective of the stage of pregnancy [36]. In addition, the interprofessional practice guidelines endorsed by the American College of Obstetricians and Gynecologists (ACOG) and the American Dental Association (ADA), recommend that pregnant women be reassured that oral health care, including the use of radiographs and local anesthesia, is safe throughout pregnancy [37, 38]. The harm of leaving active dental infections in pregnant patients may outweigh the benefits of not providing immediate dental care. Prompt treatment of dental disease before conception and during pregnancy not only benefits the expectant mother but can also prevent infant dental caries by reducing the maternal cariogenic bacterial load, and hence, the transmission of oral bacteria from mothers to children [39].

Our study also reiterates the importance of Andersen’s predisposing (education) and enabling factors such as access to free governmental health care, income, and dental health insurance in pregnant women’s access to dental care [40]. Women with lower educational attainment had a higher prevalence of toothache before pregnancy and a greater need for dental extraction before and during pregnancy. Therefore, increasing women’s awareness of the importance and safety of oral healthcare during pregnancy and integrating it with general healthcare can enhance their oral health practices and increase their access to dental care [41]. The Saudi Central Board for Accreditation of Healthcare Institutions (CBAHI) mandates the administration of well-structured preventive dental education for pregnant women in PHCs (Dental and Oral Health Standard 5.3). Although our current health system provides free dental care for Saudi citizens, our results showed that most (80%) women who went for dental care went to private dental clinics because they were unable to receive the necessary dental services at the PHCs. However, the cost of dental care and the lack of dental insurance caused others to forgo dental care. The fact that patients forgo dental care and are exposed to significant costs when they seek care underlines the need for action. Having dental insurance increases the percentage of services delivered to pregnant women. An analysis of the 2017 Medicaid (a public health insurance program for people with low income) dental claims data of pregnant women in Virginia, USA, reported 77.5% treatment, 50.4% preventive, and 93.6% diagnostic dental services among pregnant women [42]. The National Health Insurance Center (NHIC] in Saudi Arabia, an upcoming health reform, will provide free insurance coverage to all Saudi citizens [43]. Hopefully, this coverage will enable women of childbearing age to access dental care during pregnancy and to continue their dental care after delivery. Difficulties in setting up dental appointments, a lack of case management services, unavailability of dentists, and malfunctioning dental units are other barriers that hinder the utilization of dental services in PHCs that need to be resolved [44].

Limitations of our study include the fundamental characteristics of cross-sectional studies. Recall bias among respondents is another limitation; however, women are unlikely to forget a visit to a dentist during pregnancy. Although under-coverage bias is a possible limitation, we do not feel that this limitation had a significant weight on the validity of our study findings. Our study’s relatively large sample size, high response (75%) and low exclusion rates (7%), and coverage of all PHCs in the city make our findings robust and credible. Although our results may not be generalizable to pregnant women who attend private prenatal clinics in Jeddah, they can be generalized to most women attending prenatal health clinics in PHCs in the country. Furthermore, we believe that our study findings are extremely useful to the international dental and obstetric community and to primary healthcare physicians.

The engagement and support of key stakeholders in the healthcare system in Saudi Arabia, whether internal or external to the Ministry of Health, are fundamental to improving oral health care for women during pregnancy and throughout their lifespan. Current health reforms in Saudi Arabia have created an opportunity to enable women of childbearing age to access and utilize basic preventive and treatment dental services in primary healthcare settings. Based on the findings of this descriptive study, several recommendations are proposed to improve the oral health and access of pregnant women to dental care. First and foremost, the education and training of oral health providers on the importance of good oral health to the well-being of expectant mothers. The inclusion of an instructional module in the curriculum of dental schools on the care and management of pregnant women is important for educating future dentists about the importance and safety of providing dental services to women during pregnancy. Another proposed recommendation is to couple every dental examination and oral health education of referred pregnant women with dental prophylaxis. Finally, key performance indicators should be used to monitor dental referrals, dental treatment plans, and dental prophylaxis for pregnant women. The contribution and collaboration of every stakeholder in the provision of oral healthcare for women of childbearing age are crucial for improving the general well-being and oral health of women and their children.

## Conclusion

The survey of postpartum women who attended antenatal care in primary healthcare centers showed that despite the increased prevalence of dental checkup visits during pregnancy compared to pre-pregnancy and post-pregnancy, all other dental services significantly declined. Increasing women’s awareness of the importance and safety of oral healthcare during pregnancy, training dental students and primary healthcare dentists in the practice guidelines for the dental management of pregnant women, and developing and monitoring key performance indicators for maternal oral healthcare are the starting steps for improving the oral health and well-being of women and their children.

## Data Availability

The data can be provided by DA upon reasonable request.

## Abbreviations

ACOG: American College of Obstetricians and Gynecologists
ADA: American Dental Association
ASTDD: Association of State and Territorial Dental Directors
CBAHI: Central Board for Accreditation of Healthcare Institutions
ECC: Early childhood caries in children
MS: Mutans streptococci
NHIC: National Health Insurance Center
PHC: Primary healthcare centers
PRAMS: Pregnancy Risk Assessment Monitoring System
